# Dislodged Bonded Molar Tube into Wound during Orthognathic Surgery

**DOI:** 10.1155/2018/6540945

**Published:** 2018-06-04

**Authors:** Tengku Aszraf Tengku Shaeran, A. R. Samsudin

**Affiliations:** ^1^Oral and Maxillofacial Surgery Unit, School of Dental Sciences, University of Science Malaysia (USM), 16150 Kubang Kerian, Kelantan, Malaysia; ^2^Sharjah Institute for Medical Research (SIMR), University of Sharjah, Sharjah, UAE

## Abstract

**Introduction:**

Dislodgement of orthodontic appliance into operation wounds may occur while performing orthognathic surgery. Its occurrence is commonly associated with bonded upper molar tube.

**Case Report:**

A 25-year-old gentleman presented with recurrent upper right vestibular abscess three months following a bimaxillary orthognathic surgery. A bonded molar orthodontic tube had dislodged into the wound during the operation. The clinical presentation initially mimics an odontogenic infection until our investigations revealed that it originated from the dislodged appliance. The abscess was drained, the wound site was explored, and the molar tube and neighbouring rigid fixation plates and screws were removed. The patient recovered well following the procedure.

**Conclusion:**

Dislodged metal orthodontic appliance in oral wound acts as a foreign body that may exert allergic reactions, infection, or inflammation. Pre- and postoperative intraoral examination of fixed orthodontic appliances including its count should be recorded in orthognathic surgery protocol.

## 1. Introduction

Patients requiring orthognathic surgery for correction of their maxillo-mandibular disharmony will also have to undergo orthodontic treatment during both pre- and postsurgical treatment phases. Tooth alignment and preparation of the future predicted occlusion are required, so that the osteotomized jaws can be easily repositioned in the surgery in order to achieve stable results. This is followed by a period of fine-tuning and maintenance of the occlusion afterwards.

Among orthodontists, the use of bonded orthodontic molar tubes has gained popularity compared to the conventional molar banding because the former are easier to place, without the need for orthodontic separator, more friendly to the periodontium, and more comfortable to the patient [1].

Banks and Mcfarlane [2] revealed that failure rates and displacement of bonded molar and banded molar are in the range of 33.7% and 18.8%, respectively. This might contribute to the higher percentage of dislodged bonded appliance in orthognathic surgery as highlighted by Godoy et al. [3]. According to them, 76.3% of dislodged orthodontic appliance associated with orthognathic are related to involve the maxillary molars, and they were the bonded rather than banded-type appliance [2].

## 2. Case Report

A 25-year-old gentleman presented to our clinic with a complaint of recurrent pain and swelling on his right cheek of three-month duration. He visited a general practitioner each time, and the condition was resolved with analgesic and antibiotics. However, his symptoms got worse and he attended our Oral Surgery Clinic for consultation.

The patient is a fit and healthy young man with no relevant medical history and no known history of allergy. Past surgical history revealed that he had underwent bimaxillary orthognathic surgery one and half year earlier in a local hospital. Although the postoperative period was uneventful, the surgical team informed him that there was a dislodged orthodontic appliance in his right cheek that must have occurred during the operation. The team explained to the patient that this accident was realized later on the next day after the surgery when the molar tube from the right maxillary second molar was found missing, and its presence was confirmed high up in the right maxillary-zygomatic buttress area shown in the postoperative X-ray image taken on the next day following the surgery. A series of further postoperative radiographs confirmed its location, lying outside the right maxillary antrum. Due to the pronounced postoperative facial oedema at that time, no attempt was made to remove the appliance. The absence of sign and symptoms during further follow-up sessions confirmed the decision to leave it in-situ with continuous clinical observation.

**Figure 1:**
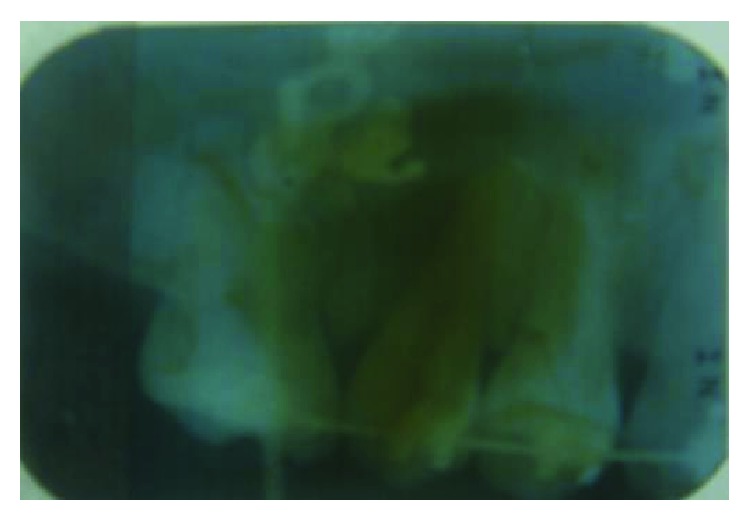
Periapical radiograph with gutta-percha (GP) in situ which had been inserted through the sinus. The GP pointing towards the area of plate and screws with the dislodged molar tube in its vicinity.

**Figure 2:**
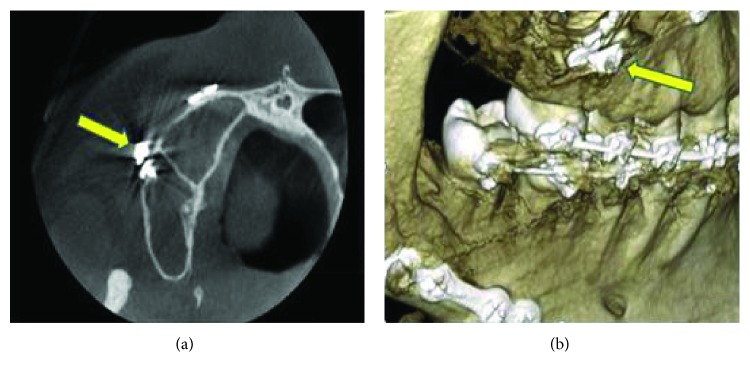
Cone Beam CT images showing the dislodged molar tube lying outside the right maxillary antrum, as indicated by the arrow in axial view (a), while its position in relation to the rigid plate and screws on the zygomatic buttress can be seen clearly in 3-D image (b).

On examination, there was no extraoral swelling noted. The mandible and maxilla seemed firm indicating good healing following previous mandibular saggital split and maxillary Le Fort I osteotomy sites and a stable class I dental occlusion. Intraorally, there was a sinus with slight pus discharge on the upper right buccal sulcus region adjacent to the upper right first premolar. All teeth in that quadrant were firm and vital. Tenderness was elicited upon palpation on the upper right vestibular region. We suspected the sinus track may originate from the dislodged appliance embedded in the cheek soft tissue. A periapical view was then taken with gutta-percha inserted into the sinus for foreign body localization purpose. The radiograph revealed the gutta-percha pointed towards the site of titanium plate and screws placed used for rigid fixation, and with the molar orthodontic tube appliance in its vicinity (Figure 1). A cone beam CT was performed to provide a 3D detailed location of the appliance (Figures 2(a) and 2(b)) and confirmed it to be located outside the maxillary antrum.

**Figure 3:**
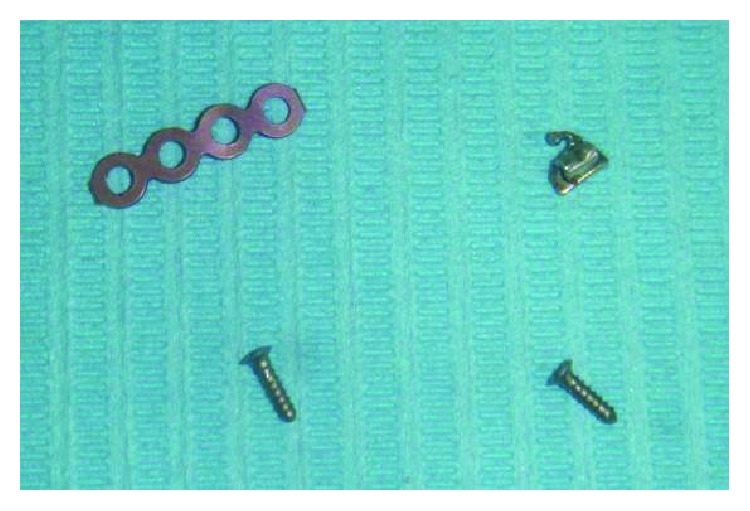
Titanium plate and screws and the stainless steel molar tube removed in the surgery.

The presence of the molar orthodontic tube foreign body reaction was suspected as the most probable cause of the recurrent right cheek pain and swelling associated with an intraoral discharging sinus. Exploration of the site was performed through the sulcular incision under general anesthesia. The dislodged molar tube was identified lying on the zygomatic bone just beneath the raised flap. It was removed by dividing some surrounding fibrous tissue strands. Just below it, one titanium straight bone plate with four screws used for fixing the previous Le Fort I osteotomy site was inspected and found to be rigidly embedded in normal bone. However, a decision was made to remove them based on the fact that they are present in an infected area. (Figure 3). The Le Fort I osteotomy site showed good healing with new bone formation. Patient had an uneventful recovery thereafter, and the orthognathic surgical team who attended him previously was informed of his progress.

## 3. Discussion

The incidence of dislodged orthodontic appliance during orthognathic surgery is rare but been recognized as one of its surgical complications. Failed orthodonthic appliances frequently occur in double jaw surgery, as in our patient who had Le Fort I and bilateral sagittal split osteotomy. It has become an accepted practice to place the wire using the cleats and hooks of molar tube or band both intraoperative and postoperatively. Intraoral manipulations during placing and removing intermaxillary fixation wire with the interim splint contribute to the appliance failure during surgery [3]. Molar tubes or orthodontic brackets are indeed a very small appliance. Hence, its displacement during orthognathic surgery may or may not be identified intraoperatively [4].

Surgeons may have different opinion with regard to the management of a dislodged orthodontic appliance. When the event occurred and notified intraoperatively, a thorough search for the foreign body till it is found is the norm, due to the fact that the dislodged orthodontic appliance is “nonsterile” and the risk of metallic ion leach deep in the tissue. However, when the foreign body is identified postsurgically, commonly during the subsequent postoperative days following routine postoperative check X-rays, there is less urge by the surgical team to search for it due to the presence of postoperative oedema, the risk of further compromising patient's airway resulting from soft tissue dissections in the exploration site, and the already drop in postoperative hemoglobin concentration, thus increasing further morbidity. The surgeon in this case had opted to leave the molar band in situ with continuous observation to minimize those morbidities since experience has taught that searching for a 4 mm size foreign body in inflamed, oedematous, and blood oozing soft tissue may take several hours!

Lammers [5] in his surgical review claimed that removal of foreign body embedded in soft tissue can be difficult and time-consuming, and the potential damage to tissues caused by the procedure must be weighed against the risk posed by a particular foreign body. He further emphasized that not all foreign bodies are discovered during the initial patient encounter [5]. Yildirim et al. investigated the diagnosis and management of retained surgical foreign bodies and recommended removal of the foreign body when identified in a symptomatic patient. However, for asymptomatic and selected cases, he supported follow-up of the patient as the treatment of choice, particularly if exploring and removing the foreign body will bring more harm to the patient [6, 7].

Despite that, the actual location of the foreign body in the face or neck also determine whether to advocate an urgent exploration or a wait and see policy. Metallic foreign bodies that have impacted into the maxillary sinus or in close proximity to major vessels or nerves or lying under the pharyngeal wall mucosa must be explored and removed in view of grave consequences. Such complications may end up with chronic maxillary sinusitis, risk of erosion and rupture of major arteries, nerve pain, and neck abscess. In the present case, the location of the molar tube high up at the zygomatic buttress, external to the maxillary sinus, seems to be less likely to cause life-threatening consequences, and it may preferably be left in situ. Reports by Teltzrow et al. and Wenger et al. supported this opinion. They found that displaced orthodontic brackets which were left in situ for longer periods had been without adverse sequelae [8, 9]. Others have reported dislodged brackets embedded into body tissues and were accidentally found later in the osseous tissue, which was theorized to be once an extraction socket [10] and in rare sites such as in the upper lip [11]. In the latter case, it was embedded following a dental trauma and remained silent for 10 years before the symptoms appeared.

On the other hand, de Queiroz et al. reported acute symptoms following loss of a bonded molar tube during orthognathic surgery [12]. The bonded maxillary second molar tube was found displaced to the inferior border of mandible in the postoperative period and a submandibular abscess developed afterwards. They advocated that the use of bonded molar tubes should be avoided in patients undergoing orthognathic surgery, as the sequelae of dislodged appliance can result in grave consequences.

Most orthodontic metal appliances such as brackets and tubes are generally made of stainless steel. They contain a mixture of iron, chromium, nickel, and a small amount of molybdenum together with small traces of other metals [13]. Despite having molybdenum in its alloy, the molar tube may still undergo corrosion and induce an inflammatory response or foreign body reaction. This is evidenced in this case by the need to divide the fibrous tissue surrounding the tube during its removal.

Preexploration localization X-ray of the molar tube demonstrated that it is positioned close to the titanium bone plates and screws which were used for rigid fixation of the Le Fort I osteotomy site. This interesting situation recalled the manufacturer's advice on cautions against mixed metals in vivo [14]. Both the titanium implants and the molar tubes, each with its own corrosion potential, can react together and produce currents, if they are in contact together in an electrochemically conductive fluid such as body fluid. In turn, this can lead to accelerated corrosion of both metals, leaching metal ions into the surrounding area, and stimulate an inflammatory or hypersensitivity response, thus producing symptoms of recurrent pain and swelling at the operated site. Macro movements of the jaw and micromotion of titanium implants and dislodged molar tube may accelerate the corrosion process. This process may have been delayed in this case due to the routine intermaxillary fixation done for six weeks following the orthognathic operation with the aim of achieving adequate bony healing at the osteotomy site to achieve a stable occlusion. The patient continued on soft diet feeding for one month following release of the intermaxillary fixation which further explains the delay in producing the symptoms of inflammation and hypersensitivity secondary to the foreign body.

## 4. Conclusion

Intraoperative surgical manipulations carry the risk of dislodging fixed orthodontic appliances during orthognathic surgery, in particular the bonded molar tube. An immediate search for the loss metal foreign body is recommended. However, when the loss is discovered postoperatively, it may be retained in situ in the wound but the length of symptom-free period can never be ascertained. It is prudent for the surgeon to perform a thorough preoperative intraoral examination on the integrity of orthodontic appliances and its count in the patient's mouth at the beginning and at the end of the surgical operation. This mandatory practice should be part of the orthognathic surgery protocol (Table 1).

## Figures and Tables

**Table 1:** Safety measures to reduce risk of appliance failure and complications.

1	Thorough examination of orthodontic appliance in patient's mouth prior to surgery and before closure of the surgical wound (appliance count and its integrity)
2	Use of molar band rather than molar tube for orthodontic treatment of patients undergoing orthognathic surgery
3	Being vigilant and cautious handling of intermaxillary fixation intraoperatively
4	Good communication with orthodontist to help prepare the patient for the scheduled surgery
